# Health-related quality of life in early-onset-scoliosis patients treated with growth-friendly implants is influenced by etiology, complication rate and ambulatory ability

**DOI:** 10.1186/s12891-019-2969-2

**Published:** 2019-12-07

**Authors:** Anna K. Hell, Lena Braunschweig, Jennifer Behrend, Heiko M. Lorenz, Konstantinos Tsaknakis, Urs von Deimling, Kiril Mladenov

**Affiliations:** 1 Pediatric Orthopaedics; Department of Trauma, Orthopaedic and Plastic Surgery, University Medical Center Goettingen, Goettingen, Germany; 2 Department of Pediatric Orthopaedics; Asklepios, Sankt Augustin, Germany; 3 Department of Pediatric Orthopaedics, Altonaer Children’s Hospital, Hamburg, Germany

**Keywords:** Health-related quality of life (HRQoL), Early-onset scoliosis questionnaire (EOSQ-24-G), Vertical expandable prosthetic titanium rib (VEPTR), Magnetic controlled growing rod (MCGR), Scoliosis, Influencing factors

## Abstract

**Background:**

Progressive Early-Onset Scoliosis (EOS) in children may lead to surgical interventions with growth-friendly implants, which require repeated lengthening procedures in order to allow adequate growth. Quality of life was studied using the validated German version of the EOS-Questionnaire (EOSQ-24-G) in surgically treated EOS children with different lengthening modalities.

**Methods:**

EOSQ-24-G and the KINDL^R^ questionnaire were given to families with EOS children who had been treated by either *vertical expandable prosthetic titanium rib* implants and repetitive lengthening surgeries every 6 months or children who had received a *magnetically expansion controlled* implant, which was externally lengthened every 3 months. Results were compared according to differences between the two tests, and with possible influencing factors such as surgical method, severity of scoliosis, relative improvement of curvature, etiology, weight, age, travelling distance, complications, ambulatory ability and others.

**Results:**

56 children with an average curve angle of 69° corrected to 33° (52%; average age 5.6 yrs) answered the EOSQ-24-G and the KINDL^R^ after an average follow-up of 3.9 years. Health-related quality of life (HRQoL) was not affected by the initial scoliosis correction, the number of surgeries or the implant type. However, there was a negative correlation with non-ambulatory status, complications during treatment and for children with a neuromuscular scoliosis.

**Conclusion:**

Using the validated EOSQ-24-G, no statistically significant differences were found between the group of children receiving repetitive surgeries and children with external lengthening procedures without surgery. However, results were influenced by the etiology, complication rate or ambulatory ability.

**Level of Evidence/Clinical relevance:**

Therapeutic Level IV

## Background

Early-Onset Scoliosis (EOS) with progressive spinal deformity of various etiologies can cause severe and complex impairment of thoracic growth and pulmonary development well known as Thoracic Insufficiency Syndrome (TIS) and therefore often requires repetitive surgical treatment during childhood and adolescence [1]. In the last two decades, several growth-friendly spinal implant systems like *vertical expandable prosthetic titanium rib* (VEPTR) implants [1, 2], *magnetically controlled devices* (MCGR) [3, 4] and others have been developed to enable control of curve progression and spinal growth, to improve lung function and to enhance quality of life for the affected children and their families. However, the majority of these implants require repetitive surgeries during childhood correlating with implant-related complications such as bacterial infections [5, 6], ossifications [7] or increased stiffness of the spine [8, 9] as well as complications during surgery and psychological distress [10].

Success of scoliosis treatment is still often measured by means of radiological parameters including reduction of spinal deformity, a low complication rate and general health of the children. Recently, psychological factors have increasingly moved into the focus of treatment. Introduction of the comprehensive questionnaire EOSQ by Corona and colleagues in 2011 allows assessment of health-related quality of life (HRQoL) specific to the target patient population of EOS children and their families [11]. Since then, the EOSQ-24 has been translated and validated in various languages [12–16] and first studies have evaluated quality of life, family satisfaction and financial burden.

The aim of this study was to evaluate quality of life in relation to different demographic data like age, body-mass-index, etiology or distance to the medical center as well as therapy-related parameters such as type of implant (VEPTR versus MCGR), complication rate or ambulatory ability. Results of the validated German version of the EOSQ-24 [17] were compared to the generic KINDL® questionnaire for children [18].

## Methods

After ethics committee approval, we conducted a cross-sectional cohort study on 56 families with children diagnosed with EOS of any etiology and associated TIS in 2018. All participants were informed about the purpose of the study and oral and written consent was obtained. During a routine follow-up, care givers of the children were asked to complete the EOSQ-24-G, the validated German version of the EOSQ-24 [11, 17], and the KINDL^R^ questionnaire, an internationally employed questionnaire for measuring the HRQoL of children without any diagnosis limitation [18]. The questionnaire was completed in the presence of a physician or the study nurse. Additionally, clinical data such as diagnosis, gender, type of treatment, age at surgical intervention, ability to walk and residence were obtained.

All participants were treated with a growth-friendly implant to control scoliotic curve progression. *Vertical expandable prosthetic titanium rib* (VEPTR) devices were implanted between the ribs and pelvis (bilateral) or ribs and lumbar spine (unilateral) with repetitive expansion surgeries approximately every six months. Magnetically controlled spinal implants (MCGR) were inserted bilateral using a VEPTR rib and pelvic fixation [19] and were expanded with an external remote controller every three months [19]. Exchange of the MCGR implants required surgery for these patients approximately every three years. After each lengthening procedure or surgical intervention, radiographs were taken and complications were documented.

Both questionnaires [17, 18] are parent-based and evaluate quality of life and burden of the disease in 24 items. Each item can be answered on a scale from one (not relevant) to five (very relevant). The score of each item was calculated as follows: averaged value of item choice - 1)/4 * 100. The mean of all 24 items is called the total score of quality of life, possibly ranging from 0 (poor) to 100 (excellent).

All obtained data including differences in HRQoL scores and impact of demographic data were analyzed statistically using Excel and if applicable with an analysis of variance (ANOVA) and *post-hoc* tests using the computer program Statistica 13.0 (Dell, USA). All data were presented as mean ± standard deviation. Statistical significance was determined as *p* < 0.05 (*), *p* < 0.01 (**) and as *p* < 0.001 (***).

## Results

### Patient demographics

Fifty six patients (24 females, 32 males) who met the inclusion criteria of treatment of Early Onset Scoliosis (EOS) with growth-friendly implants were approached. The EOSQ-24 was completed by the caregivers of all 56 children, and 54 of them (96%) also answered the KINDL^R^ questionnaire. Both questionnaires were returned with a minimum of missing data (below 2%).

The participants’ characteristics are summarized in Table 1. The most common etiology was neuromuscular scoliosis (*n* = 31), followed by congenital scoliosis (*n* = 13), syndromic scoliosis (*n* = 10) and idiopathic scoliosis (*n* = 2). Mean age at time of questionnaire was 9.2 years. At that time, all patients had undergone surgical implantation of VEPTR (*n* = 27) or MCGR (*n* = 29) devices, six patients of them had a conversion surgery from VEPTR to MCGR with their initial fixation anchors kept. Average time since implantation was 3.6 years (range 0.5–9.0) with an average of 7.4 surgical lengthening procedures for the VEPTR patients and 10.7 outpatient lengthening procedures (plus 4.2 surgical extensions if VEPTR therapy was applied before) for the MCGR patients. Study subjects were also classified according to the distance of their residence to the medical center, which was on average 218 km driving distance (range 13–545 km).

**Table 1:** Overview of patient characteristics

Variable	Value
Number of patients	56
Female	24
Male	32
Diagnosis	
Neuromuscular	31
Congenital	13
Syndromic	10
Idiopathic	2
Type of implant	
VEPTR	27
MAGEC	29
Therapy	
Average age at time of implantation in years (range)	5.6 (0.9–13.1)
Average age at time of questionnaire in years (range)	9.2 (4.9–17.1)
Number of lengthenings at time of questionnaire (range)	9.9 (2–25)

### Assessment of health-related quality of life (HRQoL)

For all evaluated parameters, the EOSQ-24-G and the KINDL^R^ questionnaire presented the same tendency. However, values for the KINDL^R^ were throughout higher than the EOSQ-24-G values. The EOSQ-24-G total score averaged at 66 (range 30–98), whereas KINDL^R^ showed an average total score of 76 (range 52–97).

Statistically, no difference could be found within the patient group regarding the following parameters: (1) sex, (2) age at time of implantation of a growth-friendly implant, (3) age at time of questionnaire completion, (4) weight, (5) type of implant, (6) distance to the medical center or (7) duration of therapy. Interestingly, also the severity of scoliosis and the relative improvement by the growth-friendly implant had no effect on subjective evaluation of quality of life. The mean scoliotic curve was 69° before and 33° directly after surgical implantation. At the time of questionnaire completion, scoliosis averaged at 39° with the relative improvement ranging from 2 to 94%.

**Fig. 1:**
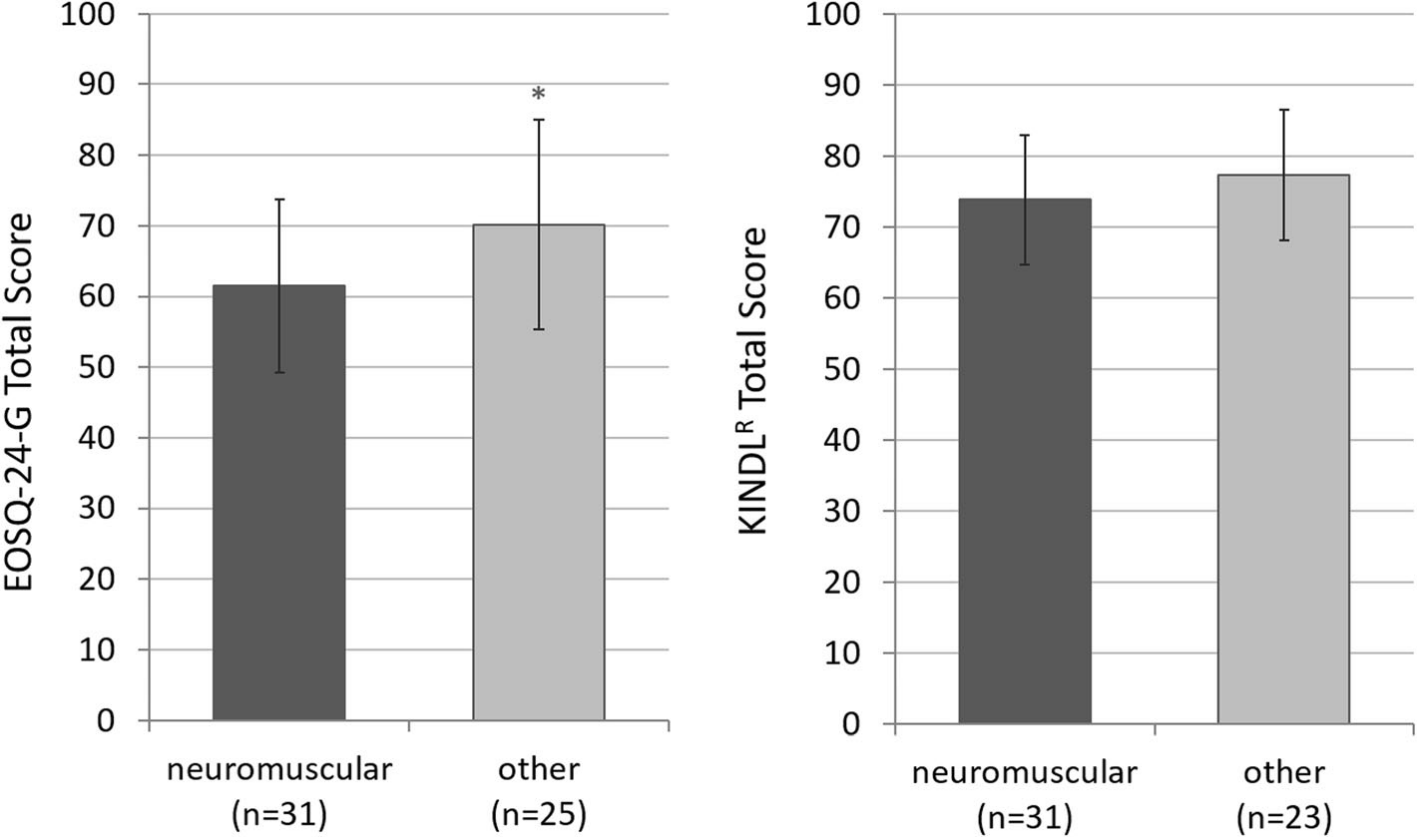
EOSQ-24-G (left) and KINDL® (right) total score in quality of life measurement of patients with neuromuscular scoliosis compared to patients with other etiologies. The means ± standard deviations are presented with statistical significance highlighted as * *p* < 0.05

The main etiology of the study group (55%) was a neuromuscular scoliosis. These patients were compared to patients with other etiologies. Neuromuscular patients had lower total scores in quality of life than patients with congenital, syndromic or idiopathic scoliosis (EOSQ-24 *p* = 0.023; KINDL^R^ *p* = 0.555) (Fig. 1).

**Fig. 2:**
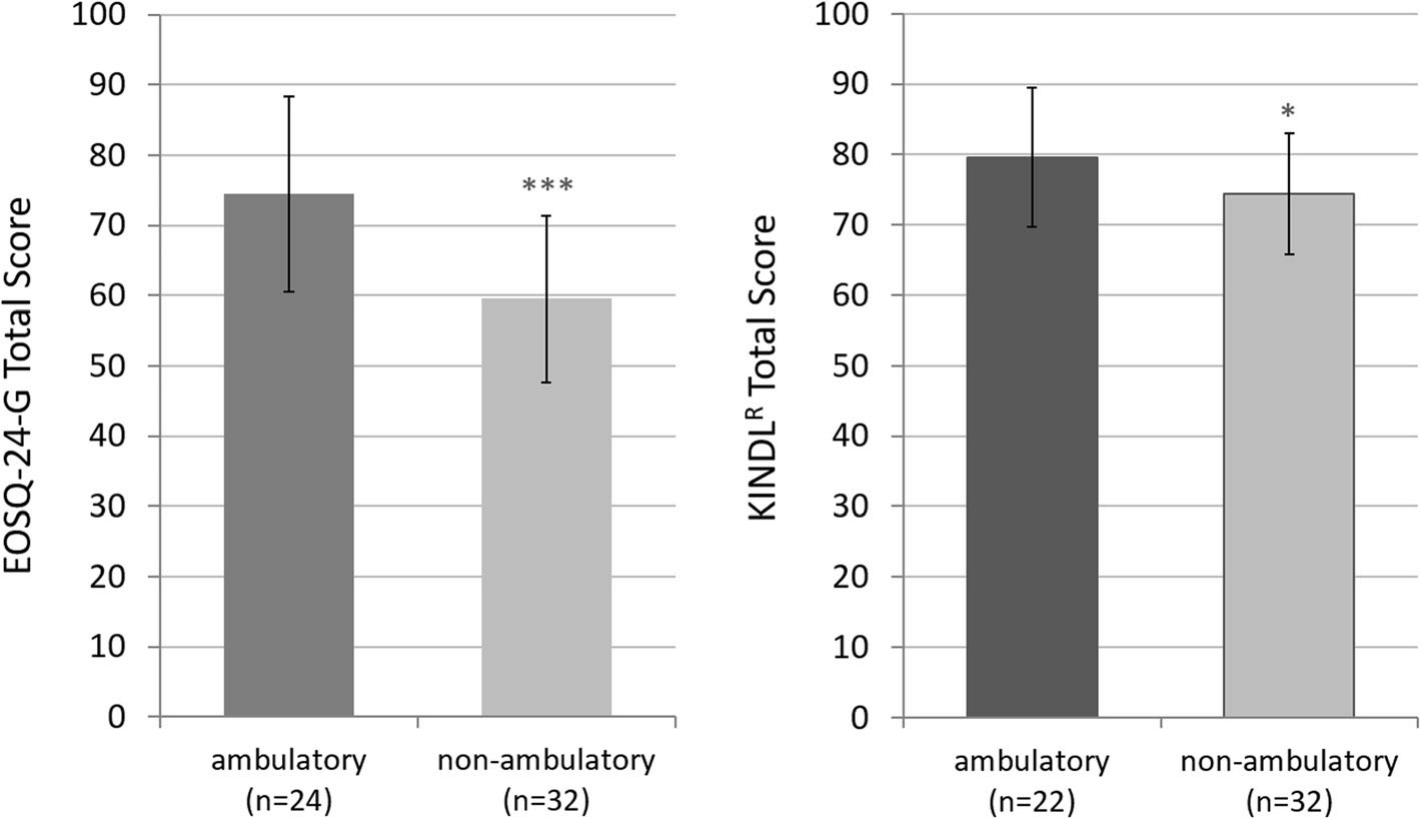
EOSQ-24-G (left) and KINDL® (right) total score in quality of life measurement of ambulatory patients compared to non-ambulatory patients. The means ± standard deviations are presented with statistical significance highlighted as *** *p* < 0.001, * *p* < 0.05

Additionally, a negative correlation of non-ambulatory patients in comparison to walking patients was found (Fig. 2), probably reflecting the fact that neuromuscular children and non-ambulatory cases presented the same patient population.

**Fig. 3:**
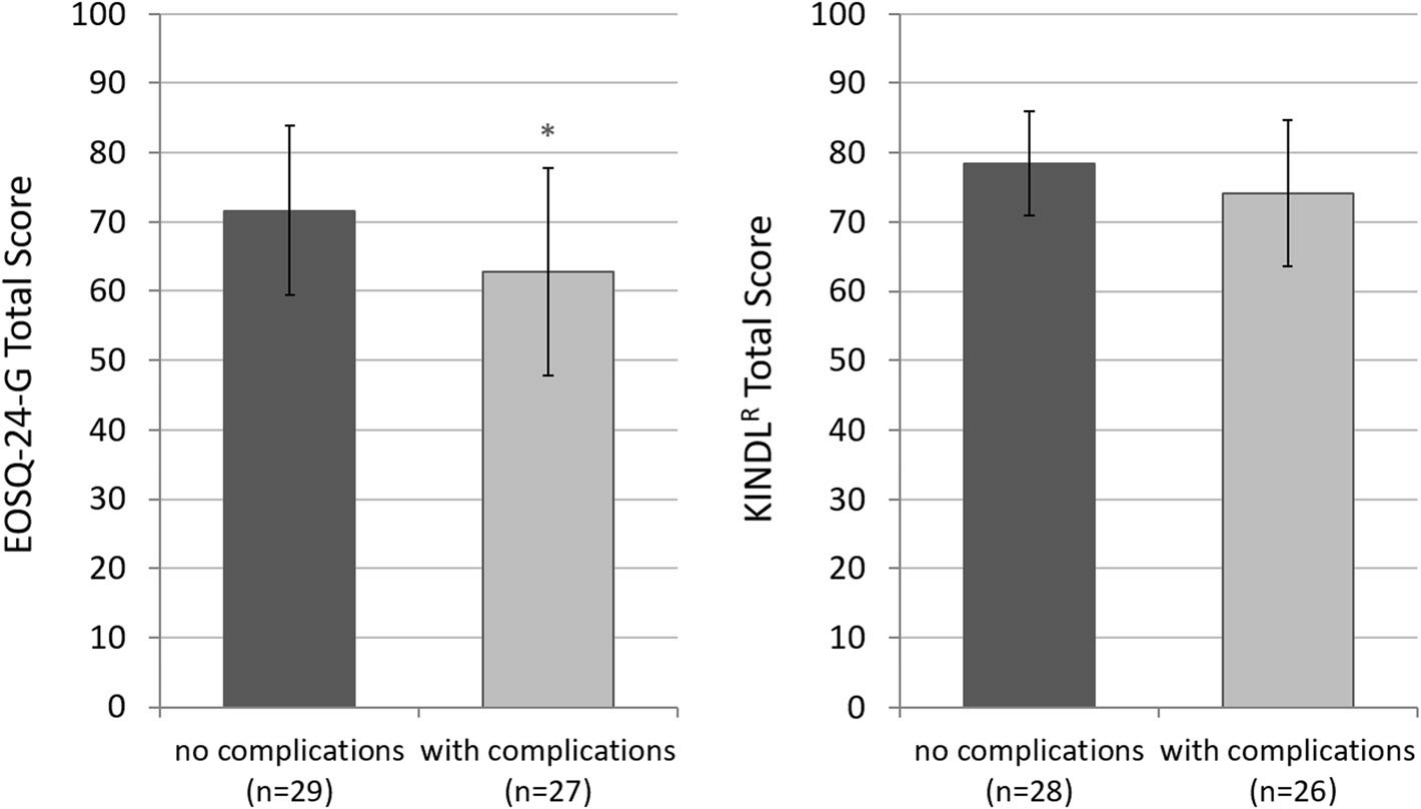
EOSQ-24-G (left) and KINDL® (right) total score in quality of life measurement of patients without complications compared to patients with any complication during therapy. The means ± standard deviations are presented with statistical significance highlighted as * *p* < 0.05

The complication rate of our study group averaged at 48% and affected VEPTR (*n* = 13/27) and MCGR (*n* = 14/29) patients likewise. HRQoL of EOS patients and their families were influenced by the occurrence of complications (Fig. 3). Study participants who had no complications during their therapy with a growth-friendly implant (52% of the total population) scored higher values than patients with at least one complication occurring during treatment (EOSQ-24 *p* = 0.020; KINDL^R^ *p* = 0.110).

## Discussion

Children with progressive EOS and their families usually face several years of continuous treatment. Most implants require repetitive lengthening procedures either surgically or externally controlled. Surgical procedures are correlated with implant-related issues such as bacterial colonization [5, 6], ossifications [7] or increased stiffness of the spine [8, 9] as well as complications during surgery, intensive care visits and psychological distress [10]. After introduction of magnetically controlled implants (MCGR) [4], a significant improvement of HRQoL was expected mainly due to the decrease in the number of surgical interventions. Interestingly, this effect could not be found in our group as well as in other patient populations [20, 21]. Only Doany et al. [22] reported on better HRQoL results in MCGR patients, but with significantly shorter follow-up in the MCGR group in comparison to patients with traditional growing rod implants. Our data showed a significantly less favorable HRQoL in non-ambulatory and neuromuscular patients as well as in children with complications. Aslan et al. [21] stated no advantage of HRQoL in MCGR children if no major complications occurred. Therefore, psychological well-being rather seems related to an uneventful treatment course no matter how many surgeries are performed than the actual number of surgical interventions, which might be less in MCGR patients with complications than in the VEPTR or traditional growing rod population.

Neuromuscular scoliosis and non-ambulatory ability were also negatively correlated to the psychosocial status most likely reflecting the overall increased care effort of these families. Many of our children were suffering from Spinal Muscular Atrophy (SMA) and had additional non-invasive ventilation as well as nutrition issues. Therefore, these additional burdens may be reflected in the less favorable results.

In a previous publication [17], the German adaption of the EOSQ-24 (EOSQ-24-G) had shown an excellent reliability in comparison to other validated translations of the EOSQ-24. In this study the EOSQ-24-G was also compared to the KINDL^R^ questionnaire, an internationally employed questionnaire for measuring the HRQoL of children without any diagnosis limitation [18]. Throughout our investigation, KINDL^R^ results were higher in comparison to EOSQ-24-G values. There was no statistical difference in both tests, meaning that both questionnaires were able to obtain identical information but on a different score value. Heterogeneity at the questionnaire evaluation time as well as no baseline data before surgery are limitations of this study.

In summary, EOSQ-24-G data reviled no differences in psychosocial data in patients and family treated with MCGR and less surgeries in comparison to VEPTR treated children with repetitive surgical interventions. Patient satisfaction was rather dependent on complications during the treatment course and the additional medical problems as seen in neuromuscular children.

## Conclusion

Health-related quality of life (HRQoL) was not dependent on the type of implant and therefore the number of surgeries. However, the etiology of neuromuscular scoliosis, complications during the therapy and non-ambulatory ability caused significant lower HRQoL values.

## Data Availability

Please contact author for data requests.

## Abbreviations

ANOVA: Analysis of variance
EOS: Early-Onset Scoliosis
EOSQ-24: Early-Onset Scoliosis -Questionnaire
HRQoL: Health-related quality of life
MCGR: Magnetic Controlled Growing Rod
TIS: Thoracic Insufficiency Syndrome
VEPTR: Vertical Expandable Prosthetic Titanium Rib
